# A Case Report: Mucinous Tubular and Spindle Cell Carcinoma of Kidney with Spindle Cell Predominance Mimicking Mesenchymal Tumour

**DOI:** 10.15586/jkcvhl.v9i4.203

**Published:** 2022-10-31

**Authors:** Natarajan Ramya, Murhekar Kanchan, Raja Anand, Sundersingh Shirley

**Affiliations:** 1 Department of Oncopathology, Cancer Institute (WIA), Adyar, Chennai, India.; 2 Department of Surgical Oncology, Cancer Institute (WIA), Adyar, Chennai, India

**Keywords:** mesenchymal tumour, mucinous tubular and spindle cell carcinoma, rare variant, renal cell carcinoma, spindle cell predominance

## Abstract

Mucinous tubular and spindle cell carcinoma (MTSCC) of kidney is a rare variant of renal cell carcinoma which was first described in the 2004 World Health Organization classification of tumours of the kidney. Morphologically, MTSCC is composed of tubules merging with bland-appearing spindle cells in a myxoid/mucinous stroma. Diverse morphological patterns have been reported in MTSCC; however, a spindle cell predominant morphology mimicking a mesenchymal tumour is rare and only two cases have been reported so far. We report a case of MTSCC with spindle cell predominance in kidney which was a diagnostic challenge. Though MTSCC usually shows an indolent course, there have been cases showing aggressive behaviour even with bland morphology. Hence, a thorough histopathological evaluation with ancillary studies are required to differentiate spindle cell predominant MTSCC from its mimics. Our case was a 40-year-old female who was incidentally found to have a well-defined hypodense lesion measuring around 2 cm in the upper pole of the right kidney. Right partial nephrectomy was performed which showed a 2.7 × 2.5 × 2 cm well-defined grey tan tumour without necrosis or haemorrhage, limited to kidney. Histopathological examination showed sheets of bland-appearing spindle cells mimicking a mesenchymal tumour. The tumour was extensively sampled, revealing small foci of tubule formation and mucinous stroma. Tumour cells were positive for CK7, AMACR, and PAX8. A final diagnosis of MTSCC was made. Hereby, we discuss ways of differentiating MTSCC from other spindle cell tumours of the kidney.

## Introduction

Mucinous tubular and spindle cell carcinoma (MTSCC) of the kidney is a variant of renal cell carcinoma (RCC), comprising less than 1% of all renal cell tumours. MTSCC is grouped under renal cell tumours in the latest 2016 WHO classification (1). Morphologically, MTSCC is composed of tubules merging with bland-appearing spindle cells in a myxoid/mucinous stroma. A review of literature reveals that MTSCC has diverse morphological patterns. MTSCC with spindle cell predominance is rare and we found two cases in literature (2, 3).

## Case history

**Figure 1:**
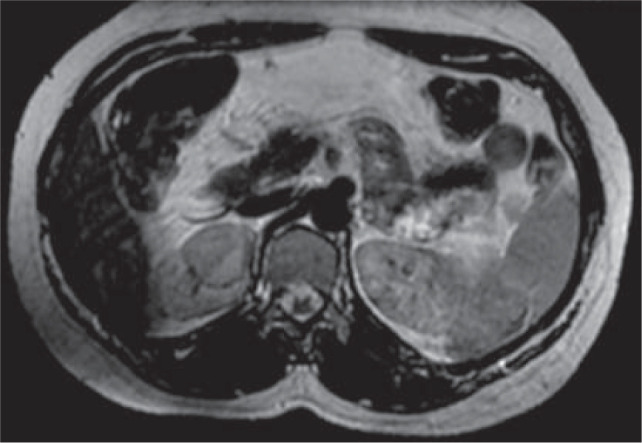
Magnetic resonance imaging: T2 isointense to intermediate well-defined lesion in the right kidney.

A 40-year-old female evaluated for polymenorrhoea was incidentally detected to have a right renal mass. She was referred to our institute for further evaluation. She had no urinary complaints and systemic examination was unremarkable. Glomerular filtration rate was reduced on the right side. CT abdomen showed a well-defined hypodense lesion, measuring 2.7 × 2.4 cm in the upper pole of the right kidney with minimal enhancement on contrast. MRI showed T1 hypointense, T2 isointense, to intermediate well-defined lesion with diffusion restriction (Figure 1). The nephrometry score was eight. The patient underwent right partial nephrectomy. No adjuvant chemoradiation was given.

**Figure 2:**
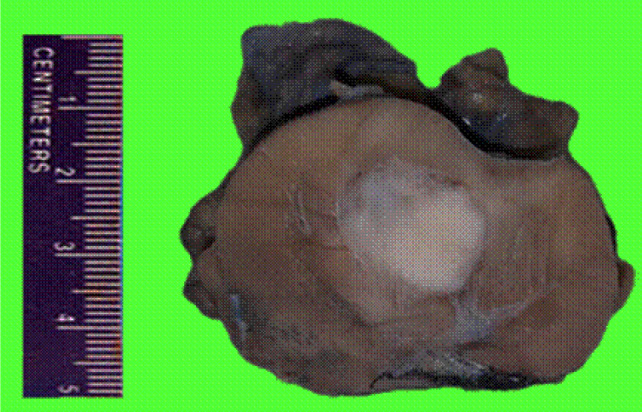
Macroscopy: Partial nephrectomy specimen showing well circumscribed grey white tumour with no haemorrhage or necrosis, limited to kidney. Adjacent kidney is unremarkable. Resected margins are free.

**Figure 3:**
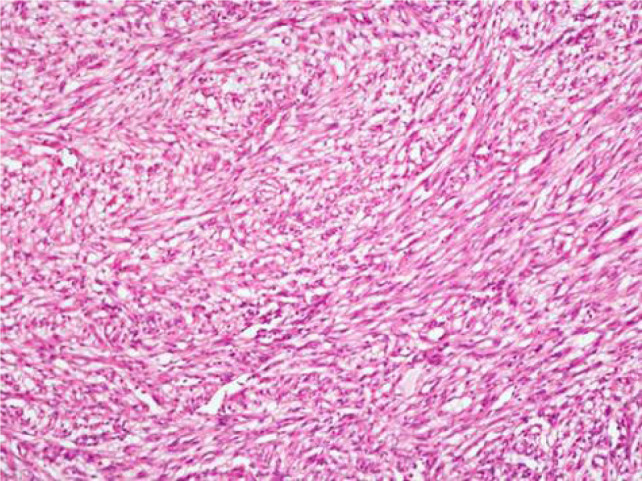
100× magnification of haematoxylin and eosin stained section of renal tumour: Sheets and fascicles of bland-appearing spindle cells.

**Figure 4:**
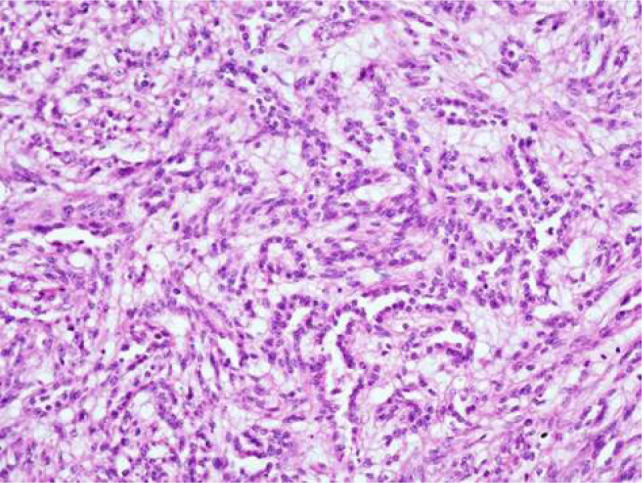
200× magnification of haematoxylin and eosin stained section of renal tumour. It shows focal tubular structures lined by bland cuboidal cells, intermingled with spindle cells, in a mucinous stroma.

**Figure 5:**
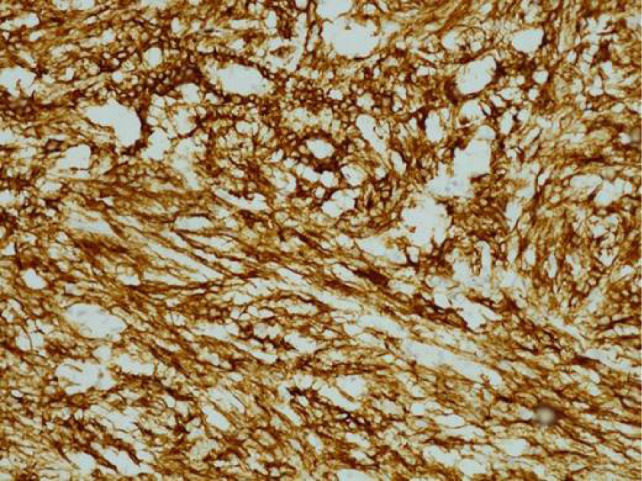
200× magnification of CK7 immunohistochemistry. Cuboidal cells lining tubules and spindle cells show positive reaction for CK7.

**Figure 6:**
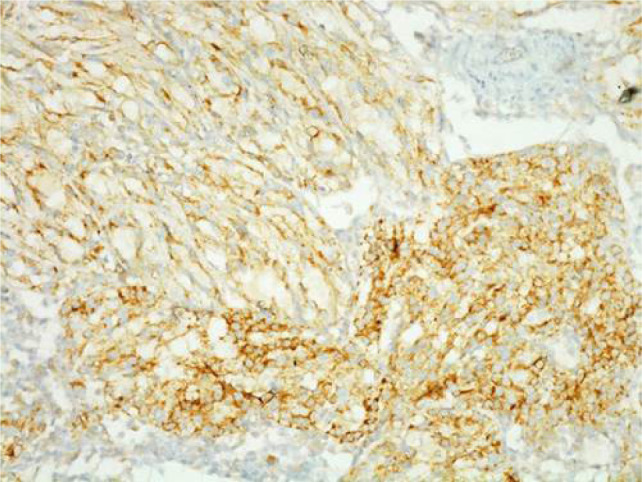
200× magnification of AMACR immunohistochemistry. Cuboidal cells lining tubules and spindle cells show positive reaction for AMACR.

Macroscopic examination showed a well circumscribed, solid, grey tumour measuring 2.7 × 2.5 × 2 cm limited to the kidney. No haemorrhage or necrosis was observed (Figure 2). Microscopic examination showed a neoplasm composed predominantly of bland-appearing spindle cells arranged in sheets and fascicles (Figure 3). The cells showed vesicular nuclei with eosinophilic nucleoli visible at 400× magnification. Mitosis was scant. No necrosis or sarcomatoid areas were seen. Patchy lymphoplasmacytic infiltrate and collection of foamy histiocytes were seen. The tumour resembled a pure mesenchymal tumour. The tumour was extensively sampled and embedded entirely which showed small foci of tubular structures lined by cuboidal cells intermingling with spindle cells (Figure 4). Alcian blue stain showed focal mucinous stroma. Provisional diagnosis of MTSCC with WHO/ISUP nuclear grade 2 was made. The TNM stage was pT1a. Immunohistochemistry showed strong and diffuse positive reaction for pancytokeratin, CK7 (Figure 5), AMACR (Figure 6), PAX8, and vimentin, and negative reaction for SMA, S100, and CD34. Ki67 was positive in 10% of tumour cells. Diagnosis of MTSCC was confirmed. The patient has no recurrence or metastasis at 24 months follow-up.

## Discussion

MTSCC is a rare variant of RCC. These tumours were initially called low-grade collecting duct carcinoma or grouped under RCC, unclassified (4). MTSCC was first described in the 2004 World Health Organization classification of tumours of the kidney. The tumour occurs predominantly in adults with age ranging from 17 to 82 years with a predilection for females. It is often an incidental finding but significant number of cases present with flank pain and/or haematuria. On CT imaging, tumour is usually heterogeneous with slight enhancement on contrast. Grossly, it is well circumscribed, solid, without areas of necrosis or haemorrhage, ranging in size from 2.5 cm to as large as 20 cm (5). Those tumours showing necrosis or infiltration are often associated with nodal and distant metastasis (6). Few tumours are cystic. Bilaterality and association with other RCC variants on the contralateral side have been reported.

Histopathology shows tubular formations lined by bland cuboidal cells, sometimes compressed to form cord-like structures. These are interspersed with fascicles of bland-appearing spindle cells. Background shows myxoid/mucinous stroma. Collection of foamy macrophages and chronic inflammatory cells are often seen. Other than these classical features, mucin-poor stroma, papillary pattern, psammomatous calcification, high-grade nuclear features, sarcomatoid transformation, neuroendocrine differentiation, and heterotopic bone formation can be seen (2, 3, 5, 6). Banyani et al. established that MTSCC originates from embryonal cell rests of impaired differentiation and that explains the tumour’s morphological heterogeneity. (7). Mucin-poor stroma and sarcomatoid transformation are associated with poor prognosis (8). Tumours with bland morphology also have shown aggressive behaviour occasionally (9, 10).

On immunohistochemistry, MTSCC shows positive reaction for AMACR, CK7, CK19, EMA, PAX8, vimentin, and E-cadherin. MTSCC is often negative for proximal tubule markers CD10 and CD15, but some cases show weak positivity. High-grade areas show p53 expression and high Ki67 index (3). Genetically, MTSCC shows losses in chromosomes 1, 4, 6, 8, 9, 13, 14, 15, and 22.

Our case showed spindle cell predominance with paucity of tubular structures which was a diagnostic challenge. Such tumours can be easily mistaken for mesenchymal tumours with entrapped renal tubules. Hence, complete sampling with extensive search for tubules, papillary structures, and high-grade areas should be done.

Differential diagnosis for MTSCC with spindle cell predominance are as follows:

1. Type I papillary RCC (PRCC) rarely shows overlapping histologic features with MTSCC-like prominent spindle cell stroma and mucin production. Certain subtle histologic features may help in distinguishing MTSCC and PRCC. MTSCC shows isolated short papillae; however, well-formed papillae with fibrovascular cores seen in PRCC are absent. Lumina of tubules are smooth in MTSCC and irregular in PRCC. Both tumours share a similar immunoprofile. Genetically, PRCC shows gains in chromosomes 7 and 17 and MTSCC shows multiple chromosomal losses (11).
2. Sarcomatoid RCC shows high-grade spindle cells with increased mitosis and necrosis. MTSCC also can show sarcomatoid transformation, but it shows adjoining typical MTSCC areas.
3. RCC with (angio)leiomyomatous stroma is an emerging entity which shows tubulopapillary structures lined by clear cells with vascular and smooth muscle stroma. The tumour is positive for CK7 and CD10, and negative for AMACR.
4. Synovial sarcoma in the kidney is usually monophasic with sheets of spindle cells. Biphasic neoplasms show epithelial structures which can be confused with tubules in MTSCC. These tumours are positive for CK, EMA, TLE1, CD99, and BCL2.
5. Angiomyolipoma that show spindle cell predominance and entrapped renal tubules mimic MTSCC. They are positive for both smooth muscle and melanocytic markers.
6. Mixed epithelial and stromal tumour of kidney can show both tubules and bland spindle cells. They are positive for ER/PR.

## Conclusions

MTSCC of the kidney shows a wide range of morphological patterns. Our case was a diagnostic challenge showing almost only spindle cells with paucity of tubular structures. Also, other RCCs, like papillary RCC, can show low-grade spindle cell foci. Hence, a thorough histopathological evaluation with ancillary studies are required to differentiate spindle cell predominant MTSCC from its mimics. The MTSCC shows an indolent course, and hence partial or radical nephrectomy would suffice, and it does not require adjuvant chemoradiation (12). However, cases showing aggressive biologic behaviour and metastasis even with bland morphology have been reported (9, 10). Hence, close follow-up is warranted (12). Prognostic significance of MTSCC with spindle cell predominance is yet to be studied.
